# Caspofungin-induced β-(1,3)-glucan unmasking is associated with enhanced innate immune clearance of *Candida (Candidozyma) auris*

**DOI:** 10.1128/spectrum.00184-26

**Published:** 2026-09-30

**Authors:** Japhet Senyo Kamasah, Bradley Edward Akin, Logan J. Baker, Adib Taba, Masoud Mahjouri-Samani, Sahar Hasim

**Affiliations:** 1 PhD Microbiology Program, Department of Biological Sciences, Alabama State University 197193, Montgomery, Alabama, USA; 2 Mercer University, Biology Department 166893, Macon, Georgia, USA; 3 Electrical and Computer Engineering Department, Auburn University 1383, Auburn, Alabama, USA; Universidade de Brasilia, Brasilia, Brazil

**Keywords:** *Candida auris*, drug resistance, cell wall remodeling, β-(1,3)-glucan exposure, macrophage phagocytosis, dectin-1, echinocandins

## Abstract

*Candida auris* is an emerging multidrug-resistant fungal pathogen that avoids early host immune detection by masking immunostimulatory β-(1,3)-glucan beneath a dense outer mannan layer. Whether caspofungin-induced cell wall remodeling can increase β-(1,3)-glucan exposure and alter innate immune interactions in *C. auris* remains poorly understood. In this study, we investigate how sublethal concentrations of caspofungin induce cell wall remodeling and modulate innate immune responses. Sublethal caspofungin concentrations were used to maintain normal growth kinetics while allowing cell wall remodeling. Caspofungin treatment increased β-(1,3)-glucan surface exposure across *Candida* strains, with the strongest response observed in *C. auris* strain 1100. This increase was supported by fluorescence microscopy, Cy3-positive cell quantification, and ImageJ-based analysis of fluorescence intensity and stained surface area. Scanning electron microscopy showed surface changes consistent with caspofungin-treated cell wall remodeling. Caspofungin-treated *Candida* cells exhibited enhanced uptake by RAW 264.7 macrophages and increased macrophage-mediated clearance at early and intermediate time points. In *C. auris*, this increased clearance persisted over 24 h, whereas the effect diminished in *C. albicans*, consistent with species-specific differences in macrophage interaction and intracellular survival. Finally, in the *Galleria mellonella* infection model, larvae infected with caspofungin-pretreated cells showed improved survival and reduced fungal burden compared with larvae infected with untreated controls. Together, these findings show that sublethal caspofungin exposure is associated with increased β-(1,3)-glucan exposure, cell wall remodeling, and enhanced innate immune clearance of *C. auris*. These results support fungal cell wall remodeling as a potential adjunct strategy to improve host-mediated control of multidrug-resistant *Candida* infections.

**IMPORTANCE:**

*C. auris* is a dangerous fungal pathogen that is difficult to treat and can evade the body’s immune defenses. One reason for its success is its ability to hide key cell wall components that immune cells normally recognize. In this study, we show that low, non-lethal levels of the antifungal drug caspofungin can alter the fungal cell wall in a way that exposes these hidden immune signals. This exposure is associated with improved recognition, engulfment, and killing of *C. auris* by immune cells. Using imaging, immune cell assays, and an infection model, we show that caspofungin-induced cell wall remodeling is associated with enhanced immune clearance and reduced fungal burden. These findings reveal an additional benefit of echinocandin treatment and suggest that antifungal drugs may help the immune system fight infection, even at doses that do not directly kill the fungus.

## INTRODUCTION

*Candida* species are the most common fungal pathogens and remain among the leading causes of hospital-acquired bloodstream infections in the United States (1, 2). The majority of infections are caused by six species: *Candida albicans*, *Candida glabrata*, *Candida tropicalis*, *Candida parapsilosis*, *Candida krusei*, and the more recently recognized *Candida auris*. Among these, *C. auris* has emerged as a serious global health threat due to its rapid global spread and persistence in healthcare settings. Since its first identification in Japan in 2009, this pathogen has rapidly spread across continents, causing outbreaks in healthcare settings. In 2022, the CDC reported 2,377 clinical cases and 5,754 screening cases of *C. auris* in the United States, reflecting its growing presence and clinical importance (3). Although recent phylogenomic analyses proposed reclassifying this organism under the genus *Candidozyma*, the nomenclature *C. auris* remains widely used for clinical clarity (4, 5).

Like other *Candida* species, *C. auris* can colonize the skin, mouth, throat, gastrointestinal tract, or vagina (6). Disease arises when colonization progresses to invasive candidiasis, enabling dissemination into deeper tissues or the bloodstream (3). These infections are often severe. An investigation of 54 *C. auris* cases reported overall mortality rates as high as 60%, with bloodstream infections reaching 68% and urinary tract infections climbing to 71% (3, 7).

A major factor that complicates the treatment of *C. auris* infections is its antifungal resistance (8–10). The three major classes of antifungal drugs used to treat systemic candidiasis, such as azoles, polyenes, and echinocandins, exhibit reduced effectiveness against this pathogen (3, 6). Surveillance studies reported that more than 90% of isolates are resistant to fluconazole (an azole), approximately 30% are resistant to amphotericin B (a polyene), and a smaller but concerning proportion are resistant to echinocandins, the current first-line therapy (3, 7). Although echinocandin resistance remains relatively uncommon overall, cases of multidrug resistance and resistance to all three antifungal classes have been documented in multiple regions (3, 6). Together, these resistance patterns highlight the urgent clinical challenge posed by *C. auris,* particularly in immunocompromised patients and individuals with underlying conditions, such as diabetes or recent surgical procedures (7, 11).

The fungal cell wall is a primary determinant of virulence and immune recognition in *C. auris*. This structure is composed of an inner scaffold of β-(1,3)-glucan, β-(1,6)-glucan, and chitin, overlaid by a dense mannan-rich outer layer (12, 13). A defining feature of *C. auris* is its ability to tightly mask β-(1,3)-glucan beneath this outer mannan layer, thereby limiting engagement of host pattern-recognition receptors, such as dectin-1 (14–16). This masking strategy is thought to contribute to reduced macrophage and neutrophil detection far more efficiently than *C. albicans*, which typically exposes β-glucan at bud scars or during hyphal growth (17). As a result, *C. auris* is often less efficiently recognized by the immune system altogether, allowing it to persist and disseminate even in immunocompetent hosts (17–19).

In *C. albicans,* the dynamic remodeling of the cell wall strongly influences host interactions. Exposure of β-glucan is associated with increased phagocytosis and cytokine production, whereas masking or compensatory chitin deposition dampens immune activation (20–24). This interplay between glucan exposure (“unmasking”) and immune recognition forms a critical conceptual framework for understanding how fungi modulate their surface structures to influence infection outcomes. Unmasking refers to the exposure of normally concealed β-(1,3)-glucan at the cell surface, which is associated with increased dectin-1-mediated recognition and macrophage responses. While this phenomenon has been extensively described in *C. albicans*, its relevance in *C. auris* remains poorly defined.

*C. auris* mannan architecture differs substantially from *C. albicans*, and glucan exposure is minimal under resting conditions (18, 19). Walker and Munro (25) reported that echinocandin treatment increased β-glucan exposure in several *Candida* species but paradoxically reduced macrophage uptake in some cases (25), likely due to compensatory increases in chitin or altered receptor engagement (26, 27).

Echinocandins, such as caspofungin, inhibit β-(1,3)-glucan synthase and weaken the structural integrity of the fungal cell wall (12, 28). At sublethal concentrations, these drugs do not inhibit growth but instead induce stress pathways that remodel the wall, potentially exposing glucan or increasing chitin deposition (25, 29). Although micafungin has been shown to produce limited or strain-dependent β-glucan exposure in *C. auris* (30), no studies to date have evaluated caspofungin specifically under sublethal conditions, nor have they connected caspofungin-induced remodeling to macrophage uptake, intracellular killing, cytokine production, or *in vivo* immune outcomes. This represents a critical gap given the central role of echinocandins in treating *C. auris* infections and the increasing frequency of echinocandin-tolerant isolates.

In addition to phenotypic variation, *C. auris* exhibits substantial genetic diversity, with four major clades distributed globally (31, 32). Whether cell wall remodeling and unmasking differ across clades or strain backgrounds remains unexplored. Understanding these distinctions is essential for predicting therapeutic outcomes, especially in settings where subtherapeutic echinocandin exposure may occur.

To address these gaps, the present study establishes sublethal caspofungin conditions for two *C. auris* strains representing distinct clades and tests whether this exposure is associated with increased β-(1,3)-glucan exposure at the cell surface. We then investigate how this remodeling is associated with innate immune responses, including phagocytic uptake, macrophage-mediated killing, pro-inflammatory cytokine production (TNF-α), and *in vivo* host survival and fungal burden using the *Galleria mellonella* infection model. By integrating structural, cellular, and organismal analyses, this work supports the conceptual model in which caspofungin-induced cell wall remodeling is associated with enhanced immune recognition and clearance of *C. auris*.

## MATERIALS AND METHODS

### *Candida* strains and growth conditions

Three *Candida* strains were used in this study: *C. albicans* strain SC5314 (Fungal Genetics Stock Center), *C. auris* strain 1100 (Clade II – East Asian), and *C. auris* strain 1103 (Clade III – African), both obtained from the U.S. Centers for Disease Control and Prevention (CDC). Strains were cultured on yeast extract–peptone–dextrose (YPD) medium composed of 10 g/L yeast extract (Thermo Scientific, Cat. No. H26769.22), 20 g/L peptone (Gibco, Cat. No. 211693), and 20 g/L D-glucose (Sigma-Aldrich, Cat. No. D9434). For solid media, 20 g/L agar (Acros Organic; CAS 9002-18-0) was added as needed.

Frozen glycerol stocks of *Candida cells* were streaked onto YPD agar plates and incubated at 30°C for 48 h. A single colony from a freshly streaked plate was used to inoculate 5 mL of YPD broth, followed by incubation at 30°C with shaking at 225 rpm for 16 h. The overnight cultures were collected by centrifugation at 3,000 × *g* for 5 min, washed three times with sterile phosphate-buffered saline (PBS), and resuspended in PBS. Washed cells were either used immediately for downstream assays or stored at 4°C for short-term use. For liquid growth assays, washed cells were adjusted to an initial optical density at 600 nm (OD₆₀₀) of 0.1 in fresh YPD broth. Cultures were incubated at 30°C under aerobic conditions unless otherwise specified.

### Caspofungin sublethal concentration determination

Sublethal concentrations of caspofungin (Sigma-Aldrich, Catalog No. SML0425) for *C. albicans* SC5314, *C. auris* 1100, and *C. auris* 1103 were determined using the broth microdilution method in sterile 96-well plates (NUNC, USA; Nest Scientific Biotechnology, Jiangsu, China). Caspofungin was reconstituted in sterile distilled water to prepare a stock solution, and working concentrations were freshly prepared in the appropriate medium before each experiment. Overnight cultures were washed and diluted in YPD broth to an OD₆₀₀ of 0.1. Twofold serial dilutions of caspofungin were prepared to achieve final concentrations ranging from 500 to 50 ng/mL. Plates were incubated at 30°C, and OD₆₀₀ was recorded hourly for 25 h using a Tecan Spark microplate reader. Growth curves were generated from triplicate wells per condition and independently repeated in three biological replicates (*n* = 3). Additional growth analyses were performed in RPMI medium to confirm that the selected concentrations remained sublethal in physiologically relevant media.

Sublethal concentrations were defined as the highest caspofungin concentrations that maintained growth kinetics comparable to untreated controls without causing growth delay or inhibition. Values were estimated from 25-h growth curves generated across serial caspofungin dilutions using a nonlinear dose-response model. Doubling times were calculated using the formula: (29). $\text{Doubling Time}=\frac{\ln(2)}{\text{Slope}}$. Values are presented as mean ± standard error (SE) and from three independent biological replicates (*n* = 3).

Based on growth-kinetic and doubling-time analyses, 50 ng/mL for *C. albicans* SC5314 and 200 ng/mL for *C. auri*s strains 1100 and 1103 were selected for subsequent experiments, as these concentrations minimally affected growth relative to untreated controls, whereas higher concentrations caused clear growth inhibition. These concentrations were therefore used in subsequent experiments to assess caspofungin-induced β-(1,3)-glucan exposure under non-growth-inhibitory conditions, consistent with previous reports showing that subinhibitory echinocandin exposure can alter *C. albicans* cell wall architecture and promote β-glucan unmasking (16, 29).

### Caspofungin treatment and β-(1,3)-glucan staining

Candida strains were grown in 5 mL YPD medium at 30°C with shaking at 225 rpm overnight. Cultures were adjusted to 0.1 OD at 600 in YPD and divided into two groups: caspofungin-treated and untreated controls. Treated cells were incubated at 30°C with shaking at 225 rpm under sublethal concentrations of caspofungin (50 ng/mL for *C. albicans* for 3 h and 200 ng/mL for *C. auris* for 4 h, respectively). Control cells (untreated) were incubated under identical conditions without caspofungin. After incubation, both treated and control cells were washed three times with PBS to minimize drug carryover before use in *in vitro* assays.

For staining, washed cells were incubated with a mouse anti-β-(1,3)-glucan primary antibody (Biosupplies Australia; Cat. No. 400-2) diluted 1:600 in 3% BSA in PBS with 1 mM MgCl₂ for 90 min at 4°C on an orbital shaker. Next, cells were washed to remove unbound primary antibody and incubated with Cy3-conjugated rabbit anti-mouse IgG (H + L) secondary antibody (Jackson ImmunoResearch; Lot No. 153631) diluted 1:300 in 5% BSA/PBS with 1 mM MgCl₂ for 45 min at room temperature in the dark with gentle agitation. Cells were then washed three times with PBS to remove excess secondary antibodies. For chitin counterstaining, after final PBS washes, cells were mixed with Calcofluor White (CFW) (Sigma-Aldrich; Pcode. 102234126) in a 1:4 (v/v) ratio immediately before imaging.

Fluorescence imaging was performed on a confocal laser scanning microscope (Nikon ECLIPSE Ti2) using a 40× objective. β-(1,3)-Glucan was detected with a Cy3/RFP filter set (excitation ~560 nm, emission ~620 nm), and chitin was visualized with a UV/DAPI filter set (excitation 405 nm, emission 480 nm). Exposure, gain, and image settings were kept constant for treated and untreated samples within each strain and channel. The treated and untreated groups were directly compared with assess changes in β-(1,3)-glucan surface exposure.

For each biological replicate (*n* = 3), Cy3- and CFW-positive cells were manually counted from 10 randomly selected, non-overlapping fields. Cy3-positive cells were defined as those showing continuous red fluorescence above the background, and CFW-positive cells as those with detectable blue fluorescence. The percent of β-(1,3)-glucan-positive cells was calculated with the formula: $\frac{\text{Cy3 positive cells}}{\text{Total CFW positive cells}}\times 100$, and the replicate value was the mean of its 10 fields.

To further quantify β-(1,3)-glucan exposure, fluorescence intensity and stained surface area were additionally analyzed using ImageJ software (v2.14.0/1.54f, National Institutes of Health, Bethesda, Maryland, USA). For each condition, images from 10 randomly selected microscopy fields per biological replicate were analyzed to determine mean fluorescence intensity and total Cy3-positive stained area. These measurements were used as supplementary quantitative indicators of β-(1,3)-glucan exposure.

### Macrophage culture and preparation

RAW 264.7 murine macrophages (ATCC, TIB-71) were maintained in Dulbecco’s Modified Eagle Medium (DMEM; Gibco) supplemented with 10% fetal bovine serum (FBS; Gibco, Catalog No. 16140071) and 1% penicillin-streptomycin (100 U/mL; Gibco). Cells were cultured at 37°C in a humidified atmosphere with 5% CO₂ in complete DMEM (cDMEM). Cells were passed when they reached ~80% confluence. For subculturing, adherent cells were detached using 0.05% Trypsin-EDTA (Gibco, Catalog No. 25300062), washed in cDMEM, and then resuspended in fresh medium. Cell density and viability were determined using a hemocytometer with trypan blue exclusion. For each experiment, macrophages were seeded at the required density for the assay and allowed to adhere overnight prior to co-culture with *Candida*. To ensure reproducibility, only low-passage cultures (<20 passages) were used. Macrophage viability was also assessed by trypan blue exclusion after 24 hours of co-culture, and viability remained above 90% under all tested conditions.

### Scanning electron microscopy (SEM)

To visualize cell surface remodeling, caspofungin-treated and untreated cells were prepared under the same conditions described for the β-(1,3)-glucan staining assay. After treatment, the fungal cells were washed three times with PBS and fixed in 2.5% glutaraldehyde overnight at 4°C. The fixed samples were washed in PBS and dehydrated through a graded ethanol series (30%, 50%, 70%, 90%, and 100%; 10 min each step). Samples were then resuspended in 100 µL absolute ethanol, and 10 µL was mounted on a slide (TED PELLA, INC; Prod No. 16111) with double-sided carbon tape, and sputter-coated with a thin layer of gold using a Quorum Q150R ES Plus sputter coater to enhance conductivity.

SEM imaging was performed at 5,000× to 20,000× magnification using a ZEISS EVO-10 scanning electron microscope under high-vacuum mode at an accelerating voltage of 10 kV. Untreated and caspofungin-treated samples were processed in parallel, with imaging parameters kept constant across all conditions to allow direct comparison. SEM was used to assess changes in cell surface morphology following caspofungin treatment; however, this method does not directly identify specific cell wall components, such as β-(1,3)-glucan, mannan, or chitin.

### Macrophage phagocytosis assay

Phagocytosis was evaluated using RAW 264.7 macrophages as previously described, with modifications (33). Macrophages were seeded at 1 × 10⁵ cells per well in sterile 96-well tissue culture plates and incubated overnight at 37°C in a 5% CO₂ incubator to promote adherence. The next day, caspofungin-treated and untreated cells were prepared as described above and adjusted to 2 × 10⁵ cells/mL. *Candida* cells were added to macrophage cultures at a multiplicity of infection (MOI) of 2 and co-incubated for 60, 90, and 150 min at 37°C in 5% CO₂ to assess different stages of phagocytosis. After co-incubation, each well was washed with sterile PBS to remove non-phagocytized *Candida* cells. Macrophages were then lysed with sterile deionized water (10 min, room temperature) to release internalized *Candida* cells. Lysates were diluted 1:100 in PBS, and 100 µL of each was plated on YPD agar in triplicate. Plates were incubated at 37°C for 48 hours. Colony-forming units (CFUs) were counted manually.

Phagocytosis was measured by counting the *Candida* cells recovered from macrophage-containing wells after macrophage lysis. Phagocytosis (%) was calculated as $\left(\frac{CFU\;from\;macrophage\;containing\;wells}{mean\;{CFU}_{control}\;wells\;without\;macrophages}\right)\times 100$. Control wells containing *Candida* cells without macrophages were included under the same assay conditions. These control wells were used to confirm that caspofungin treatment did not affect fungal recovery during the assay. All conditions were performed with three technical replicates and three independent biological replicates (*n* = 3).

### Macrophage killing assay

Macrophage-mediated killing of *Candida* cells was assessed using a modified macrophage killing assay described by Zhang et al. (33). RAW 264.7 macrophages were plated at 1 × 10⁵ cells per well in 96-well tissue culture plates and incubated overnight at 37°C with 5% CO₂ to allow cell adherence. The next day, non-adherent cells were gently removed by aspirating the medium and rinsing with sterile 1× PBS. Caspofungin-treated and untreated cells were added to macrophage monolayers at an MOI of 2 and co-incubated under standard culture conditions for 90 min to ensure maximal phagocytic uptake. After this initial incubation, the medium and non-engulfed fungal cells were removed by washing the wells three times with sterile PBS. Fresh cDMEM medium was then added, and the macrophages with engulfed *Candida* culture were incubated for an additional 2, 6, and 24 h to evaluate the killing process.

At each time point, macrophages were lysed by adding sterile deionized water for 10 minutes at room temperature to release intracellular *Candida* cells. The entire contents of each well were collected, diluted 1:100 in PBS, and 100 µL of each dilution was plated onto YPD agar plates in duplicate. Plates were incubated at 37°C for 48 h, and colony-forming units (CFUs) were counted to measure fungal survival. For each strain and treatment group, parallel control wells containing *Candida* cells without macrophages were included under the same assay conditions to measure fungal survival in the absence of macrophage activity and to confirm that caspofungin treatment did not affect fungal recovery during assay processing.

Percent killing for each well was calculated using the formula: $\%\;\text{killing}=\left(1-\frac{CFU\;from\;macrophage\;containing\;wells}{mean\;{CFU}_{control}\;wells\;without\;macrophages}\right)\times 100$, where CFU from macrophage-containing wells represents the number of surviving *Candida* cells recovered after co-culturing with macrophages, and the mean CFU control represents the average recovery of the same *Candida* strain under the same caspofungin treatment condition but without macrophages. All conditions were performed with three technical replicates and three independent biological replicates (*n* = 3).

### TNF-α (alpha) detection assay

RAW 264.7 macrophages were seeded at a concentration of 1 × 10⁵ cells/well in a tissue culture-treated 24-well plate (Thermo-Fisher) and incubated overnight at 37°C in a humidified incubator with 5% CO₂ to allow adherence. The next day, culture media were gently aspirated and replaced with fresh cDMEM containing UV-killed *C. albicans* SC5314, *C. auris* 1100, or *C. auris* 1103 cells at an MOI of 2. UV treatment was used to inactivate fungal cells and minimize the effect of live fungal replication on TNF-α production, allowing macrophage responses to be assessed in response to fungal cell wall exposure. Both untreated and caspofungin-treated cells were included for each strain. Co-cultures were incubated for 4 h at 37°C with 5% CO₂. After incubation, the supernatants were collected and passed through a 0.22-µm Ultrafree-CL GV filter (UFC40GV0S, Merck Ltd., Japan) to remove cell debris and uninternalized fungal material. Filtered supernatants were assayed for TNF-α using the Invitrogen Mouse TNF-α ELISA Kit (Catalog # BMS607-3TEN) according to the manufacturer’s protocol. Absorbance was measured at 450 nm using a microplate reader. Blanks were subtracted, and TNF-α concentrations were calculated using the standard curve generated from the ELISA kit. Macrophage-only wells were included as negative/background controls. All treatments were performed in triplicate for each biological replicate, and the experiment was independently repeated three times (*n* = 3).

### *Galleria mellonella in-vivo* survival assay

*In vivo* virulence of *C. albicans* SC5314, *C. auris* 1100, and 1103 was assessed using the *Galleria mellonella* infection model. Untreated and caspofungin-treated cells were washed and resuspended in sterile PBS to a final concentration of 1 × 10^7^ cells/mL. For each strain and treatment group, 10 μL of suspension (≈approximately 1 × 10⁵ cells) was injected into the last left proleg of individual larvae (Carolina Biosciences, Catalog #: 143928) using a Hamilton syringe. Control larvae were injected with 10 μL PBS alone. Each experimental group consisted of 10 larvae, and all experiments were performed as three independent biological replicates.

After injection, larvae were transferred to sterile Petri dishes containing *Galleria food* and incubated at 35°C in the dark. Survival was monitored for 7 days, and data were recorded every 24 h. Larvae were considered dead when they showed no movement after being gently touched with a pipette tip.

### *Galleria mellonella* fungal burden assay

Fungal burden was determined following infection with untreated or caspofungin-treated *Candida* strains as described for the survival assay above. At 6, 24, and 48 h post-infection, six larvae from each experimental group were randomly selected for fungal burden determination. Individual larvae were transferred into separate wells of a sterile 12-well plate containing 1 mL sterile PBS and mechanically homogenized. Homogenates from the six larvae within each experimental group were pooled to reduce biological variability between individual larvae and generate a representative group-level fungal burden. Serial dilutions were prepared in sterile PBS, and 100-µL aliquots from appropriate dilutions were plated in technical triplicate on YPD agar plates. Plates were incubated at 30°C for 24 h, after which colony-forming units (CFU) were enumerated. Fungal burden was calculated as CFU per larva, log₁₀-transformed, and expressed as Log₁₀ (CFU per larva). PBS-only controls were processed in parallel to confirm sterility and absence of contamination. All experiments were performed in three independent biological replicates, each with three technical replicates. Each biological replicate consisted of an independently performed infection experiment using a separate batch of six larvae and freshly prepared *Candida* inocula. Statistical comparisons between untreated and caspofungin-treated groups for each strain and time point were performed using Welch’s *t*-test.

### Statistics

All experiments were performed with three biological replicates and three technical replicates per condition, unless otherwise noted. Data from replicates (*n* = 3) were analyzed in GraphPad Prism v10 and are presented as mean ± SD. Error bars in figures represent the standard deviation across all replicates. Values for caspofungin growth analyses were estimated using nonlinear regression with a four-parameter dose-response model. Statistical significance was defined as *P* < 0.05. Group comparisons were evaluated using one-way or two-way ANOVA with Tukey’s or Šídák’s post-hoc tests, depending on the design. Survival and burden data from *Galleria mellonella* assays were analyzed using Kaplan–Meier survival curves with log-rank (Mantel–Cox) tests and Welch’s *t*-test, respectively.

## RESULTS AND DISCUSSION

### Determined sublethal concentration for *C. albicans and C. auris* strains

**Fig 1:**
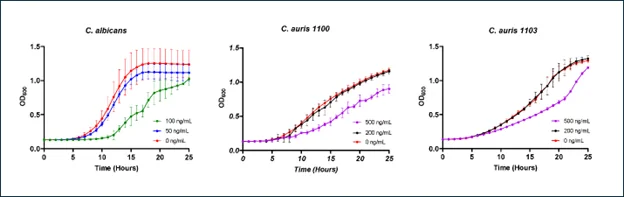
Growth kinetics of *Candida* species in response to varying concentrations of caspofungin. Overnight cultures were diluted to an OD of 0.1 and seeded in 96-well plates containing YPD broth and different concentrations of caspofungin. Concentrations were serially diluted from 500 to 50 ng/mL. Growth was monitored by measuring the optical density at 600 nm (OD₆₀₀) over a 25-h period. Cultures were exposed to different caspofungin concentrations. Growth of *C. albicans SC5314* was significantly reduced at concentrations ≥100 ng/mL. In contrast, *C. auris* strains 1100 and 1103 exhibited minimal growth impairment at lower concentrations, with substantial growth inhibition observed at concentrations ≥500 ng/mL. These growth profiles were used to define sublethal caspofungin concentrations for subsequent experiments. Data are expressed as mean ± SD from three independent biological replicates, each performed with three technical replicates.

To identify sublethal caspofungin concentrations that preserve overall growth while enabling downstream cell wall analyses, we monitored the growth kinetics of *C. albicans* SC5314 and *C. auris* strains 1100 and 1103 over 25 h (Fig. 1; Table 1). Sublethal concentrations were defined as the highest caspofungin concentration that maintained growth kinetics comparable to untreated controls without statistically significant growth delay or inhibition. Growth curves were generated from three independent biological replicates, with triplicate technical wells per condition, and doubling times are presented as mean ± SE.

**TABLE 1:** Doubling times of *Candida* strains exposed to caspofungin in YPD medium^*a*^

*Candida* strains	Caspofungin concentration (ng/mL)	Mean doubling time (h ± SE)	Adjusted *P*-value vs control
*C. albicans*	0	2.713 ± 0.130	NA^*b*^
	50	2.707 ± 0.120	0.9991
	100	15.72 ± 0.040	0.0002
*C. auris* 1100	0	3.527 ± 0.078	NA
	200	4.090 ± 0.643	0.6780
	500	7.253 ± 0.080	<0.0001
*C. auris* 1103	0	4.720 ± 0.070	NA
	200	4.900 ± 0.068	0.2387
	500	6.720 ± 0.100	0.0002

^*a*^ Values are presented as mean doubling time in hours ± SE from three independent biological replicates. Adjusted *P*-values indicate comparisons with untreated controls within each strain.

^*b*^ NA, not applicable.

For *C. albicans*, growth inhibition became apparent at concentrations ≥100 ng/mL, with doubling time increasing from 2.713 ± 0.13 h (untreated) to 15.72 ± 0.04 h at 100 ng/mL (Fig. 1; Table 1). At 50 ng/mL, *C. albicans* maintained growth kinetics comparable to those of untreated controls, with only a modest increase in doubling time (2.707 ± 0.12 h). Therefore, this concentration was selected as the sublethal caspofungin concentration for *C. albicans* in subsequent assays. This finding is consistent with previous reports demonstrating that low-concentration echinocandin exposure preserves growth while inducing cell wall stress and remodeling in *C. albicans* (25, 27, 29).

In contrast, both *C. auris* strains showed increased tolerance to caspofungin across the same concentration range. For strain 1100, doubling times remained nearly unchanged between untreated cells (3.527 ± 0.078 h) and those exposed to 50–200 ng/mL of caspofungin (4.09 ± 0.643 h). Similarly, strain 1103 showed minimal variation, with untreated cells doubling at 4.720 ± 0.07 h compared with 4.90 ± 0.068 h under exposure to 50–200 ng/mL of caspofungin. Growth inhibition in both strains was observed at higher concentrations ≥500 ng/mL (Fig. 1 and Table 1). Based on this growth profile and following the same sublethal selection strategy established for *C. albicans*, 200 ng/mL was chosen as the highest non-growth-inhibitory concentration for *C. auris* strains.

To confirm that the selected concentrations remained sublethal under physiologically relevant media conditions, additional growth analyses were performed in RPMI medium. Both *C. albicans* and *C. auris* exhibited increased tolerance to caspofungin in RPMI compared with YPD, indicating that the YPD-derived concentrations used in this study represent a conservative sublethal selection and remain non-growth-inhibitory under both experimental conditions (Fig. S1 and Table S1). These RPMI data reduce the possibility that the macrophage phenotypes observed later were due to caspofungin-induced growth defects at the selected concentrations.

Together, these findings indicate that *C. auris* maintains growth at higher caspofungin concentrations than *C. albicans* under these conditions, consistent with species-specific differences in echinocandin susceptibility.

### Caspofungin-induced β-(1,3)-glucan unmasking visualized by immunofluorescence

**Fig 2:**
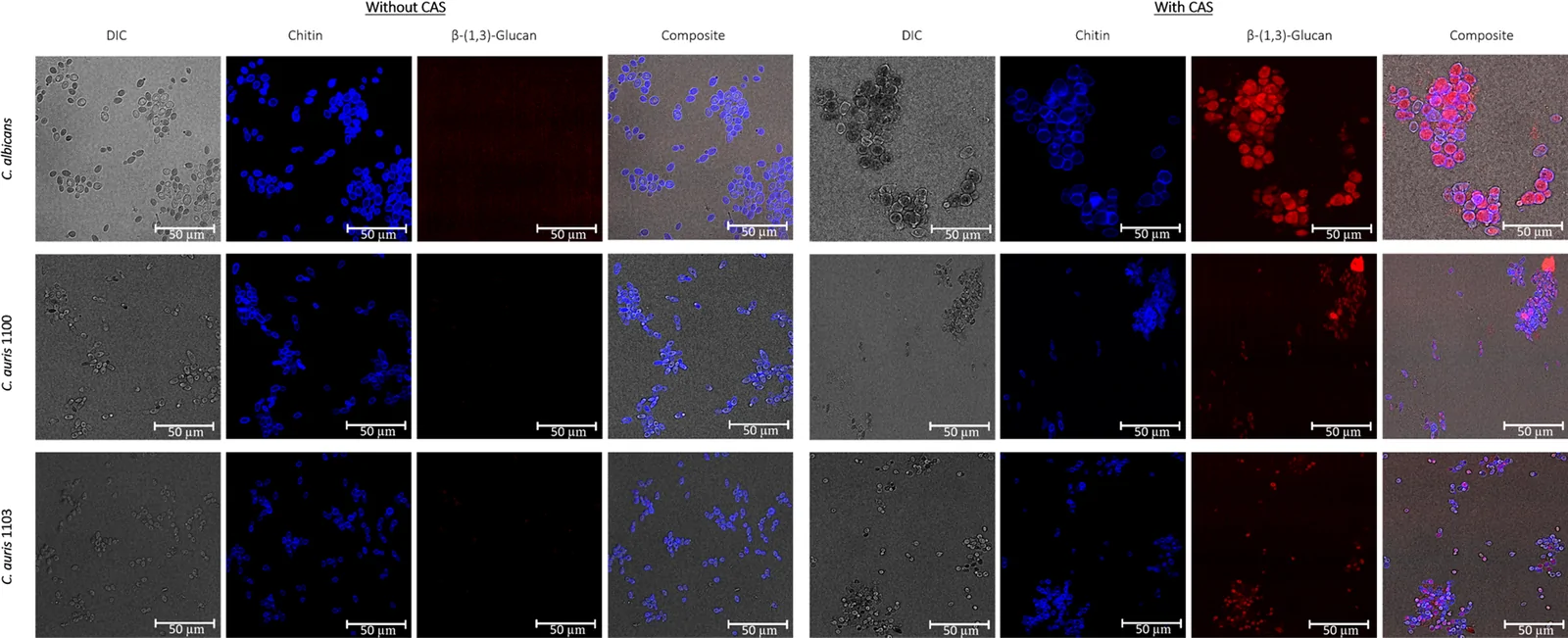
Immunofluorescence of β-(1,3)-glucan on the cell surface of *Candida* strains. Representative fluorescent micrographs of *C. albicans* and *C. auris* strains 1100 and 1103 under untreated and sublethal caspofungin treatment. *Candida* strains were subcultured in YPD broth containing caspofungin along with the untreated controls for 3 h at 50 ng/mL for *C. albicans* and 4 h at 200 ng/mL for *C. auris*. Untreated cells are labeled as “without CAS,” and caspofungin-treated cells are labeled as “with CAS.” Chitin and septa were stained with Calcofluor white (CFW, blue). CFW was mixed in a 1:4 v/v ratio prior to imaging. β-(1,3)-Glucan was detected using a mouse anti-β-(1,3)-glucan primary antibody, followed by a Cy3-conjugated secondary antibody (red). Primary antibody staining was performed at a 1:600 dilution in PBS with 3% BSA and 1 mM MgCl_2_ for 90 min. Secondary antibody staining was performed at a 1:300 dilution in 5% BSA/PBS with 1 mM MgCl_2_ for 45 min. Fluorescence imaging was performed using a Nikon ECLIPSE Ti2 confocal laser scanning microscope with a 40× objective. β-(1,3)-Glucan was detected with a Cy3/RFP filter set (excitation ~560 nm, emission ~620 nm), and chitin was visualized with a UV/DAPI filter set (excitation 405 nm, emission 480 nm). Images are representative of three independent biological replicates. Scale bar = 50 µm.

To determine whether sublethal caspofungin treatment increased β-(1,3)-glucan exposure in *Candida*, we performed dual staining with Calcofluor White (CFW, blue) to visualize chitin-rich cell wall structure and Cy3-labeled antibodies (red) to detect β-(1,3)-glucan under untreated (w/o CAS) and caspofungin-treated (w CAS) conditions (Fig. 2). Differential interference contrast (DIC) imaging was included to visualize cell morphology under each condition and to compare untreated and caspofungin-treated cells. In untreated controls, all strains showed intense CFW staining with low-level and localized Cy3 staining, consistent with basal β-(1,3)-glucan exposure at septal regions, indicating that β-(1,3)-glucan was largely masked by the outer mannan layer. After caspofungin treatment (50 ng/mL for *C. albicans* and 200 ng/mL for both *C. auris* strains), we observed an increase in cell surface-associated Cy3 signal of the cells. This effect was consistent across biological replicates, supporting the idea that sublethal caspofungin exposure induces cell wall remodeling in a manner that makes β-(1,3)-glucan more accessible at the cell surface.

**Fig 3:**
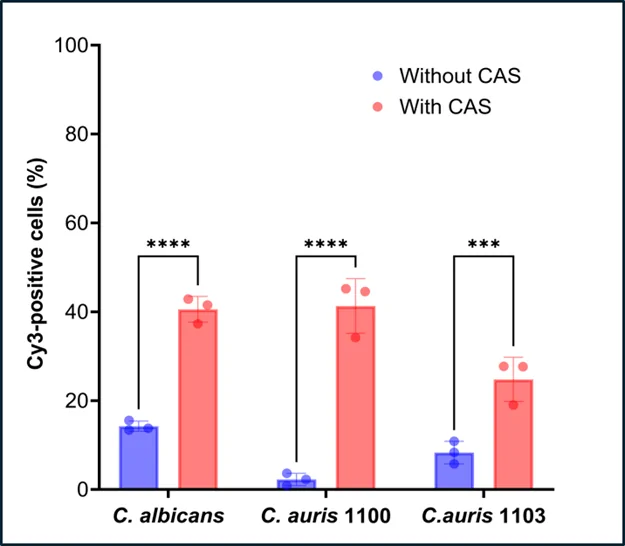
Quantification of caspofungin-induced β-(1,3)-glucan exposure in *Candida species*. β-(1,3)-Glucan exposure was quantified by Cy3 staining in *C. albicans* SC5314 and *C. auris* strains 1100 and 1103 under untreated conditions (without CAS, blue) or following sublethal caspofungin treatment (with CAS, red). β-(1,3)-Glucan staining was performed by adding the primary mouse antibody at a 1:600 dilution in PBS with 3% BSA and 1 mM MgCl_2_ for 90 min. The secondary antibody was added at a 1:300 dilution in the 5% BSA/PBS with 1 mM MgCl_2_ for 45 min. Each dot represents the mean percentage of Cy3-positive cells quantified from 10 random microscopy fields for a single biological replicate (*n* = 3). Bars represent the mean ± SD. Caspofungin-treated cells showed significantly higher β-(1,3)-glucan exposure compared with untreated controls within each strain. Statistical analysis was performed using one-way ANOVA within each strain, followed by Tukey’s multiple-comparisons test on replicate means (****P < 0.0001; ***P < 0.0005).

The increase in red fluorescence was most pronounced in *C. auris* strain 1100, which displayed strong Cy3 intensity distributed across the entire cell surface, followed by strain 1103, which showed patchy but distinct exposure patterns. *C. albicans* exhibited discrete Cy3 signals concentrated along septal rims and bud scars, consistent with previously reported echinocandin-induced remodeling in this species (34, 35). To quantify these observations, we calculated the percentage of Cy3-positive cells across 10 randomly selected, nonoverlapping fields per biological replicate, with three independent biological replicates analyzed per condition (Fig. 3). Caspofungin pretreatment significantly increased β-(1,3)-glucan exposure in all strains compared with untreated controls. For *C. albicans*, the proportion of Cy3-positive cells increased from approximately 12% in untreated cultures to about 42% after treatment (****P < 0.0001). *C. auris* strain 1100 showed the most notable increase, rising from roughly 3% untreated to approximately 41% after caspofungin exposure (****P < 0.0001). Strain 1103 also exhibited an increase from around 8% to 26% (***P < 0.0005).

To further assess β-(1,3)-glucan exposure as a continuous variable rather than a binary positive/negative outcome, fluorescence intensity and stained surface area were additionally quantified using ImageJ. Images from 10 randomly selected fields per biological replicate were analyzed for mean Cy3 fluorescence intensity and total Cy3-positive stained area. Both measurements confirmed increased β-(1,3)-glucan exposure following caspofungin treatment across all strains, supporting the qualitative microscopy observations and percentage-based quantification (Fig. S2).

Together, the microscopy and quantitative fluorescence data show that sublethal caspofungin is associated with increased β-(1,3)-glucan exposure in both *C. albicans* and *C. auris*. At the species level, *C. auris* exhibits minimal basal β-(1,3)-glucan exposure but responds strongly to caspofungin treatment, and at the strain level, *C. auris* 1100 shows the most pronounced increase in exposure, whereas strain 1103 displays a more limited response. Although the extent of β-(1,3)-glucan exposure varies among strains, the overall trend indicates that caspofungin induces cell wall remodeling that increases the accessibility of β-(1,3)-glucan at the cell surface. Because mannan organization was not directly measured in this study, these data support increased β-(1,3)-glucan exposure but do not directly define the specific changes in mannan distribution.

Interestingly, our findings differ from those reported by Horton et al*.* (31), who observed limited and strain-dependent β-(1,3)-glucan exposure in *C. auris* following subinhibitory micafungin treatment. Several factors may contribute to this difference. First, the *C. auris* strains used in the present study differ from those examined by Horton et al., and strain-to-strain variability in echinocandin responsiveness has been previously described in *C. auris* (36, 37). In addition, although micafungin and caspofungin are both echinocandins that target β-(1,3)-glucan synthase, differences in drug structure may influence the degree of cell wall stress and downstream remodeling induced by each compound (29, 34, 35). Finally, methodological differences between the studies may also have contributed to the differing observations, including variation in how sublethal drug concentrations were selected and how β-(1,3)-glucan exposure was measured and quantified. Although the extent of β-(1,3)-glucan exposure varies among strains, the overall trend indicates that caspofungin treatment increases the accessibility of β-(1,3)-glucan at the cell surface, consistent with drug-induced cell wall remodeling. Consistent with this interpretation, prior studies by Walker and colleagues and Lee and colleagues show that alterations in chitin content and organization can disrupt cell wall architecture and expose β-(1,3)-glucan that is normally concealed beneath the outer mannan layer in untreated cells (38, 39).

### Visualization of *Candida* cell wall remodeling by scanning electron microscopy

**Fig 4:**
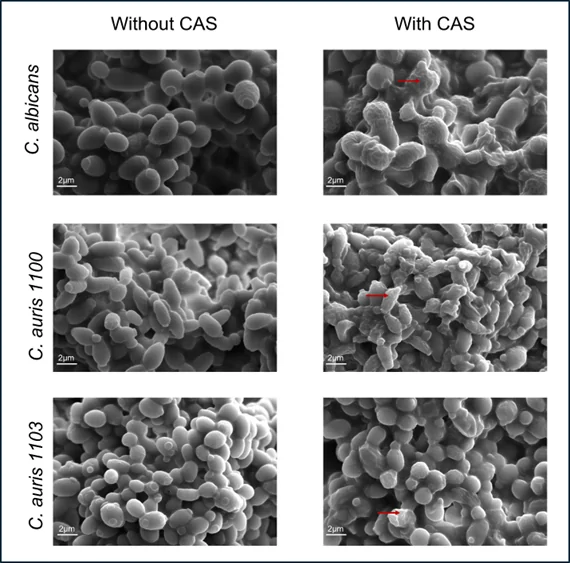
Scanning electron microscopy reveals caspofungin-induced alterations in *Candida* cell surface morphology. SEM micrographs show the surface morphology of *C. albicans* SC5314 and *C. auris* strains 1100 and 1103 under untreated conditions (without CAS) and after exposure to caspofungin (with CAS). Cells were treated with sublethal caspofungin concentrations of 50 ng/mL for *C. albicans* SC5314 and 200 ng/mL for *C. auris* strains 1100 and 1103 before SEM imaging. After treatment, the cells were washed then fixed in 2.5% glutaraldehyde. The fixed samples were washed in PBS and dehydrated through a graded ethanol series. Samples were then dried and mounted on a slide with double-sided carbon tape, and sputter-coated with a thin layer of gold using a Quorum Q150R ES Plus sputter coater to enhance conductivity. SEM imaging was performed at 5,000× to 20,000× magnification using a ZEISS EVO-10 scanning electron microscope under high-vacuum mode at an accelerating voltage of 10 kV. Untreated cells display smooth and intact cell surfaces. In contrast, caspofungin-treated cells exhibit altered surface morphology, including surface irregularities, surface indentations, and wrinkling (red arrows), consistent with caspofungin-associated changes in cell surface morphology under sublethal conditions. Scale bar = 2 µm.

After confirming β-(1,3)-glucan exposure by immunofluorescence microscopy (Fig. 2 and 3), we next examined whether caspofungin treatment was associated with visible surface-level changes by scanning electron microscopy (SEM). Caspofungin-treated and untreated cells were prepared under the same treatment conditions and imaged by SEM. Untreated cells from all strains display smooth, rounded surfaces with intact outer cell walls, consistent with normal yeast morphology. In contrast, caspofungin-treated cells exhibited visible surface-level changes, including surface irregularities, indentations, and wrinkling (Fig. 4). While SEM cannot directly identify specific cell wall components, such as chitin, mannan, or β-glucan, we interpret these images as evidence of altered surface morphology rather than direct confirmation of cell wall architectural disruption. These SEM findings are consistent with caspofungin-associated cell surface remodeling and complement the fluorescence-based evidence of increased β-(1,3)-glucan exposure. These changes appeared more pronounced in *C. albicans* and *C. auris* strain 1100, which demonstrated the strongest increase in β-(1,3)-glucan exposure.

### Macrophage phagocytosis enhancement

To better understand macrophage interaction with untreated and caspofungin-treated *Candida* cells, we performed a quantitative phagocytosis assay using RAW 264.7 macrophages at the 90-min co-incubation time point. Following incubation, non-phagocytosed fungal cells were removed by washing, macrophages were lysed, and the number of intracellular cells was quantified by CFU recovery, providing a measure of phagocytic uptake. The 90-min time point was selected because it provided measurable fungal uptake while still allowing comparison between untreated and caspofungin-treated groups.

**Fig 5:**
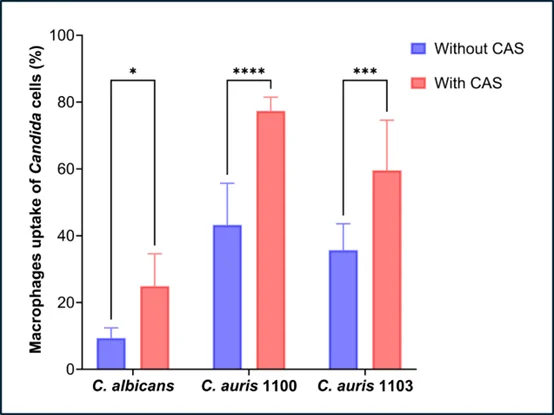
Caspofungin treatment enhances uptake of *Candida* cells by RAW 264.7 macrophages. *C. albicans* and *C. auris* strains (1100 and 1103) were treated with 50 and 200 ng/mL caspofungin, respectively. RAW 264.7 macrophages (1 × 10⁵ cells/well) were co-incubated with untreated (without CAS; untreated) or the caspofungin-treated (with CAS; treated) *Candida* cells at an MOI of 2 for 90 mins. After incubation, macrophages were lysed using sterile deionized water. Lysates were diluted in PBS and plated onto YPD agar for CFU enumeration. Macrophage uptake was quantified using the following formula: Phagocytosis (%) = [CFU from macrophage-containing wells/mean CFU from matched Candida-only control wells without macrophages] × 100. Caspofungin-treated cells exhibited significantly higher engulfment compared with untreated controls across all strains. Data are presented as mean phagocytosis (%) ±SD from three independent biological experiments (*n* = 3). Statistical analysis was performed using one-way ANOVA with Šídák’s multiple comparisons test (*P < 0.05, ***P < 0.001, ****P < 0.0001).

As shown in Fig. 5, *C. albicans* phagocytosis increased by 15.57% (*P* = 0.0273). The effect was stronger in *C. auris* 1100, which showed a 34.14% increase (*P* < 0.0001), with phagocytosis rates of ~77.38% in caspofungin-treated cells versus ~43.23% in untreated controls; *C. auris* 1103 showed a 23.88% increase (*P* = 0.0005), with ~59.56% uptake in caspofungin-treated cells versus ~35.69% in untreated cells. These results show that caspofungin treatment enhances macrophage recognition and uptake of both *C. albicans* and *C. auris*, with the strongest effect observed with strain 1100 (Fig. 5).

These findings highlight the role of caspofungin in shaping the interaction between *Candida* and host immune cells. Caspofungin alters the fungal cell wall and increases surface exposure of β-(1,3)-glucan. In untreated cells, lower β-(1,3)-glucan exposure may limit macrophage recognition compared with caspofungin-treated cells. The increased phagocytic uptake observed at 90 minutes correlated with increased β-(1,3)-glucan exposure in caspofungin-treated cells; however, the present study does not directly establish that glucan exposure alone is responsible for this phenotype. Other caspofungin-induced cell wall changes may also contribute to the increased macrophage uptake observed in this assay. The species and strain-specific differences observed here suggest variability in the degree of glucan masking and in the extent of remodeling triggered by caspofungin.

However, it remains unclear whether caspofungin-treated *Candida* cells engulfed by macrophages at 90 minutes are ultimately killed or can escape. To address this question, we performed macrophage-killing assays as described in the following section.

### Caspofungin-treated *Candida* cells show increased macrophage-mediated clearance

Macrophage killing was evaluated by co-incubating RAW 264.7 cells with untreated and caspofungin-treated *Candida* strains, then lysing the macrophages with sterile water at defined time points (2, 6, and 24 h), and plating the lysates to measure the surviving fungal CFUs. Parallel *Candida*-only control wells without macrophages were included for each strain and treatment condition to account for fungal survival and recovery during water-lysis processing. Percent killing was calculated relative to the matched *Candida*-only control for the same strain and caspofungin condition.

**Fig 6:**
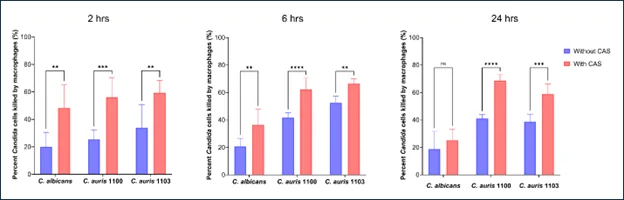
Caspofungin treatment is associated with increased macrophage-mediated clearance of *Candida* species. Macrophage-mediated clearance of *C. albicans* SC5314 and *C. auris* strains 1100 and 1103 were measured following co-incubation with RAW 264.7 macrophages. *Candida* cells were added to macrophages at an MOI of 2, and phagocytosis was allowed to proceed for 90 minutes. Extracellular *Candida* cells were removed by washing. Fresh DMEM was added to the wells, and co-cultures were allowed to continue for 2, 6, and 24 h. At each time point, macrophages were lysed with sterile deionized water, and lysates were diluted 1:100 in PBS and plated on YPD agar for CFU recovery. Matched *Candida*-only control wells without macrophages were included for each strain and treatment condition, and processed under the same conditions. Percent killing was calculated as: % killing = [1 − (CFU from macrophage-containing wells / mean CFU from matched control wells without macrophages)] × 100. Red bars represent caspofungin–treated cells (with CAS), and blue bars represent untreated cells (without CAS). Data are presented as mean ± SD from three independent biological experiments. Statistical significance was determined using two-way ANOVA followed by Šídák’s multiple comparisons test. Significance levels are defined as follows: ns, not significant; **P < 0.01; ***P < 0.001; ****P < 0.0001.

At 2 h (Fig. 6), caspofungin-treated cells showed higher levels of killing than untreated controls for all strains. In *C. albicans*, macrophage killing increased from approximately 25% in untreated cells to nearly 50% in caspofungin-treated cells. For *C. auris* 1100, killing was about 30% in untreated cells compared with over 65% in caspofungin-treated cells, while strain 1103 showed around 35% killing in untreated cells versus approximately 60% in caspofungin-treated cells. These findings indicate that caspofungin-treated cells showed increased macrophage-mediated clearance during the early stages of interaction across both species. These results suggest that increased early macrophage-mediated clearance is associated with increased β-(1,3)-glucan exposure, which may contribute to more efficient macrophage recognition and antifungal activity.

At 6 h, caspofungin-treated cells continued to show greater killing than untreated controls (Fig. 6). In *C. albicans*, killing increased from 40% in untreated cells to nearly 55% in caspofungin-treated cells. For *C. auris* strain 1100, killing was ~35% in untreated cells compared with over 55% in caspofungin-treated cells. Strain 1103 showed a similar pattern, with ~30% killing in untreated cells versus almost 45% in caspofungin-treated cells. These results indicate that the increased macrophage-mediated clearance of caspofungin-treated cells was sustained beyond the early stage. As reported in prior studies, sustained macrophage-mediated killing at intermediate time points is consistent with prolonged antifungal responses following β-(1,3)-glucan exposure, which may involve mechanisms, such as nitric oxide production and pro-inflammatory cytokine signaling (40). Although increased β-(1,3)-glucan exposure correlated with enhanced macrophage uptake and killing in our study, the present work does not directly establish a mechanistic relationship between glucan exposure and macrophage recognition. Therefore, these data should be interpreted as an association between caspofungin treatment, increased β-(1,3)-glucan exposure, and enhanced macrophage-mediated killing rather than direct proof that β-(1,3)-glucan exposure alone caused the killing phenotype. Additional mechanistic studies will be needed to determine the extent to which β-(1,3)-glucan exposure specifically contributes to the observed immune response, as other caspofungin-induced cell wall changes may also play a role.

At 24 h, increased macrophage-mediated clearance persisted for *C. auris* strains but not for *C. albicans* (Fig. 6). In *C. auris* 1100, killing increased from 40% in untreated cells to nearly 70% in caspofungin-treated cells. For strain 1103, killing was ~35% in untreated cells compared with more than 55% in caspofungin-treated cells. In contrast*, C. albicans* showed no clear difference between untreated and caspofungin-treated cells at this late time point. This species-specific outcome likely reflects differences in survival strategies between species. *C. albicans* can form hyphae that facilitate escape from macrophages and reduce susceptibility to intracellular killing, which may explain the convergence of untreated and caspofungin-treated cells by 24 h. In contrast, *C. auris*, which possesses limited filamentation capacity (41), remains inside macrophages and is continuously exposed to oxidative bursts, nutrient deprivation, and degradative enzymes, making these cells more vulnerable to macrophage-mediated killing during intracellular residence.

Together, these findings show that caspofungin-treated *Candida* cells exhibited increased macrophage-mediated clearance at early and intermediate time points. This increase correlated with increased β-(1,3)-glucan exposure, but the data do not exclude contributions from other caspofungin-induced cell wall changes or form differences in initial macrophage uptake.

### Caspofungin-treated *Candida* cells increase TNF-α secretion by macrophages

**Fig 7:**
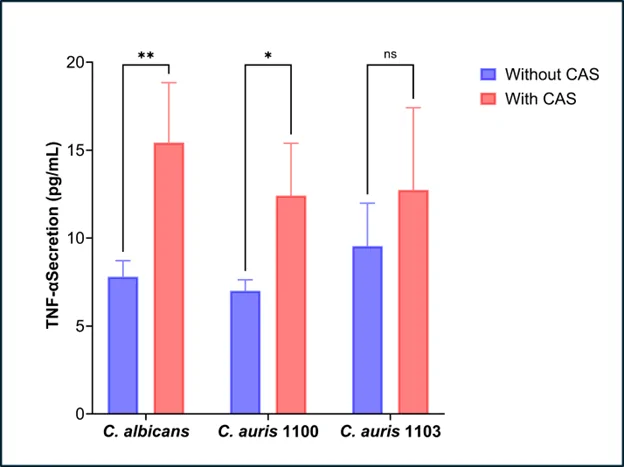
Caspofungin-treated *Candida* cells increase TNF-α secretion by macrophages. RAW 264.7 macrophages were co-incubated with untreated (without CAS, blue) or caspofungin-treated (with CAS, red) *Candida* cells at an MOI of 2. Supernatant was collected after 4 h of co-incubation, filtered through a 0.22-µm filter, and assayed for TNF-α by ELISA according to the manufacturer’s instructions. Absorbance was measured at 450 nm, and secreted TNF-α levels are shown as pg/mL. Macrophages exposed to caspofungin-treated *C. albicans* and *C. auris* strain 1100 showed significantly higher levels of TNF-α compared with untreated controls, with no significant difference observed for *C. auris* strain 1103. Bars represent mean ± SD from three biological replicates. Statistical analysis was performed using one-way ANOVA within each strain, followed by Tukey’s multiple comparisons test (*P < 0.05, **P < 0.01; ns, not significant).

To test whether caspofungin-induced unmasking influences cytokine signaling, we measured TNF-α secretion from macrophages during co–culture with UV-killed untreated or caspofungin-treated *Candida* using a sandwich ELISA (Fig. 7). UV-killed cells were used to minimize the effects of fungal replication and to evaluate macrophage responses in relation to fungal cell wall exposure.

Macrophages exposed to caspofungin-treated *C. albicans* secreted ~5.2 pmol/L TNF-α compared with ~1.45 pmol/L in the untreated group. A similar increase was observed for *C. auris* strain 1100, with TNF-α increasing from ~1.2 pmol/L in the untreated group to ~3.5 pmol/L in the caspofungin-treated group. In contrast, *C. auris* strain 1103 showed a smaller, non-significant increase from ~2.0 pmol/L in the untreated group to 3.2 pmol/L in the caspofungin-treated group.

Increased β-(1,3)-glucan exposure in caspofungin-treated cells was associated with increased TNF-α secretion by macrophages. TNF-α is a key pro-inflammatory cytokine produced by activated macrophages and serves as an indicator of heightened innate immune signaling (42, 43). The weaker TNF-α response observed with *C. auris* strain 1103 suggests strain-specific differences in cell wall remodeling and immune stimulation. Overall, these findings indicate that caspofungin-treated *Candida* cells can increase macrophage cytokine responses, but this response should be interpreted as an association with caspofungin-induced cell wall remodeling rather than direct proof that β-(1,3)-glucan exposure alone caused the increase in TNF-α secretion.

### Caspofungin pretreatment of *Candida* cells is associated with improved host survival in the *Galleria mellonella* infection model

**Fig 8:**
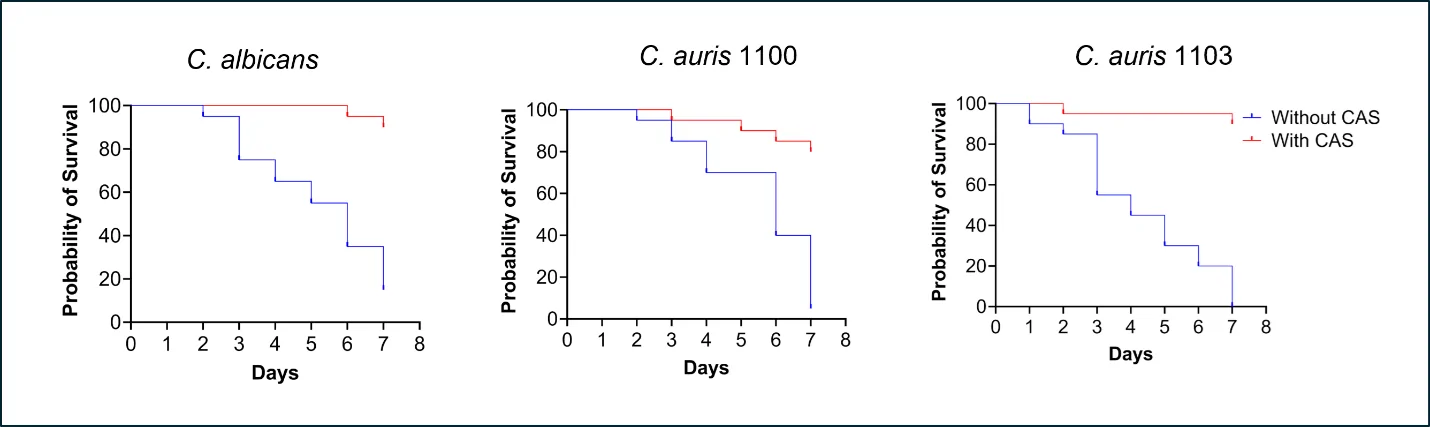
Caspofungin-treated *Candida* cells are associated with improved survival of *Galleria mellonella* larvae. Kaplan–Meier survival curves of *G. mellonella* larvae infected with untreated (without CAS, blue) or caspofungin-treated (with CAS, red) *C. albicans* SC5314 and *C. auris* strains 1100 and 1103. Larvae were infected in the lower left proleg by delivering 10 μL of suspension (approximately 1 × 105 cells). Larvae were transferred to a sterile petri dish containing food, and survival was monitored for 7 days. Larvae were considered dead when they showed no movement after physical stimulation. Infection with caspofungin-treated cells was associated with significantly improved larval survival compared with untreated controls for all strains. Statistical significance was assessed using the log-rank (Mantel–Cox) test (*P* < 0.0001 for all comparisons). Each group contained 10 larvae, and experiments were performed in three independent biological replicates.

To evaluate the effect of caspofungin on host survival, we performed Kaplan–Meier analyses in *G. mellonella* infected with our *Candida* cells. Larvae were injected with caspofungin-treated or untreated cells and monitored daily for survival over 7 days. In all cases, as shown in Fig. 8, the untreated groups showed a rapid decline in survival, with mortality beginning as early as day 2 and reaching 60%–80% by day 7. In contrast, larvae infected with caspofungin-treated cells exhibited significantly prolonged survival. These findings show that caspofungin pretreatment was associated with improved host survival *in vivo* across the tested *Candida* strains.

Although *C. auris* exhibits substantial variability in antifungal resistance across strains, echinocandins remain the primary first-line treatment for invasive infections (3). Our data indicate that sublethal exposure to caspofungin does not inhibit fungal growth under the conditions tested but is associated with cell wall remodeling and improved host survival. This is in line with prior reports showing that caspofungin induces remodeling of β-glucan and chitin, which is associated with enhanced immune recognition (25–27).

Interestingly, the magnitude of protection varied slightly among strains. Strain 1103 showed the strongest survival benefit, suggesting that genetic background may influence the extent of drug-induced remodeling and subsequent immune engagement. However, because this study did not directly test the mechanism of protection in the *Galleria* model, these strain-specific survival differences should be interpreted as being associated with caspofungin pretreatment rather than directly caused by β-(1,3)-glucan exposure alone. This observation is consistent with previous findings of strain-dependent variability in echinocandin responses (19).

Taken together, these results support the potential of caspofungin not only as a therapeutic agent but also as a modulator of host–pathogen interactions through drug-induced cell wall remodeling. By altering fungal cell wall architecture and increasing β-(1,3)-glucan exposure, caspofungin treatment may support improved host-mediated control of infection beyond direct growth inhibition. These findings underscore the importance of further investigating echinocandin-induced cell wall remodeling and β-(1,3)-glucan exposure in *C. auris* as a strategy to improve immune-mediated control of infection.

### Caspofungin-treated *Candida* cells show reduced fungal burden in *Galleria mellonella*

**Fig 9:**
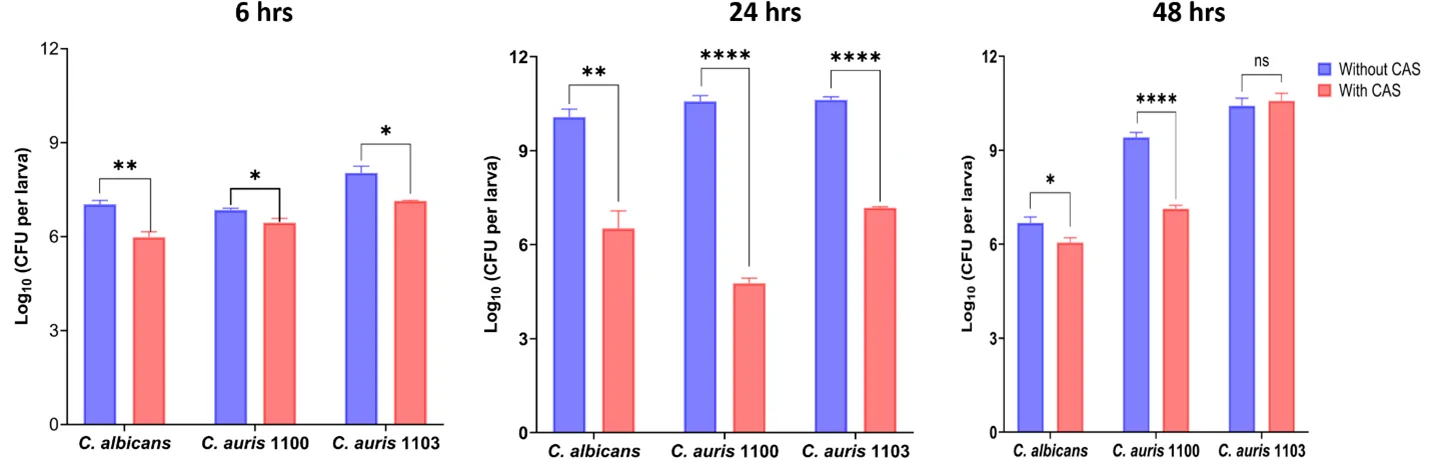
Caspofungin-treated *Candida* cells are associated with reduced fungal burden in *Galleria mellonella*. Larvae were infected with approximately 1 × 10^5^ untreated (without CAS, blue) or caspofungin-treated (with CAS, red) *C. albicans* SC5314 and *C. auris* 1100 or *C. auris* 1103 cells. Infected larvae were incubated at 37°C, and fungal burden was quantified at 6, 24, and 48 h post-infection. Data are presented as Log₁₀ (CFU per larva) from three independent biological replicates. For each biological replicate, six larvae (*n* = 6) per group were pooled, homogenized, plated in technical triplicate, and values were averaged prior to analysis. Bars represent mean ± SD. Statistical comparisons between untreated and caspofungin-treated groups within each strain and time point were performed using Welch’s *t*-test. *P < 0.05, **P < 0.01, ***P < 0.001, ****P < 0.0001; ns, not significant.

Fungal burden was assessed by infecting *G. mellonella* larvae with caspofungin-treated and untreated *Candida strains*. The infected larvae were homogenized and plated for CFU enumeration at 6, 24, and 48 h post-infection. The Log₁₀ CFU/larva values, fold reductions, and statistical significance for each strain and time point are summarized in Table 2 and shown in Fig. 9.

**TABLE 2:** Fungal burden following infection with untreated or caspofungin-treated *Candida* strains in *Galleria mellonella*^*a*^

Time post-infection	Strain	Log₁₀ CFU/larva		Approximate fold reduction
		Untreated	Caspofungin-treated	
6h	*C. albicans*	7.034	5.979	11
	*C. auris* 1100	6.849	6.448	2.5
	*C. auris* 1103	8.035	7.143	8
24h	*C. albicans*	10.07	6.517	3.5 × 10^3^
	*C. auris* 1100	10.576	4.772	6.4 × 10⁵
	*C. auris* 1103	10.627	7.181	2.8 x 10^3^
48h	*C. albicans*	6.678	6.058	4
	*C. auris* 1100	9.414	7.135	190
	*C. auris* 1103	10.42	10.579	No reduction (ns)

^*a*^ Values represent Log₁₀ CFU per larva from the *Galleria mellonella *fungal burden assay. Approximate fold reduction was calculated by comparing untreated and caspofungin-treated groups after converting Log₁₀ CFU values to CFU values. “No reduction” indicates that the caspofungin-treated group did not show reduced fungal burden compared with the untreated control at that time point.

At 6 h post-infection**,** larvae infected with caspofungin-treated cells exhibited a significant reduction in *Candida* burden across all strains (Fig. 9). The most pronounced fungal burden reduction was observed *in C. albicans* (**P < 0.01), with caspofungin-treated cells showing 5.979 Log₁₀ CFU/larva compared with 7.034 Log₁₀ CFU/larva in untreated controls, corresponding to an approximately 11-fold reduction. A modest but statistically significant decrease was observed in larvae infected with *C. auris* strains 1100 and 1103 (*P < 0.05). In *C. auris* 1100, caspofungin-treated cells showed 6.448 Log₁₀ CFU/larva compared with 6.849 Log₁₀ CFU/larva in untreated controls, with an approximately 2.5-fold reduction. Similarly, *C. auris* 1103 showed an approximately eightfold reduction in fungal burden following caspofungin treatment, decreasing from 8.035 Log₁₀ CFU/larva in untreated controls to 7.143 Log₁₀ CFU/larva in caspofungin-treated cells. The early reduction in fungal burden of all three strains is consistent with increased β-(1,3)-glucan exposure observed *in vitro*, but these data do not directly prove that β-(1,3)-glucan exposure alone caused the improved fungal clearance. These findings align with previous reports showing that echinocandin treatment can expose fungal β-glucan and influence innate immune recognition (8, 10). Because *G. mellonella* relies on innate immune defenses, these results are consistent with improved host-mediated fungal clearance following caspofungin pretreatment.

At 24 h, the reduction in fungal burden was more pronounced across all strains. Significantly reduced fungal burden was observed for all strains, with particularly strong effects in *C. auris* 1100 and *C. auris* 1103 (****P < 0.0001), and a robust reduction in *C. albicans* (**P < 0.01). In *C. albicans*, caspofungin-treated cells showed 6.517 Log₁₀ CFU/larva compared with 10.070 Log₁₀ CFU/larva in untreated controls, corresponding to an approximately 3,500-fold reduction. The most pronounced reduction was observed in *C. auris* 1100, where caspofungin-treated cells showed 4.772 Log₁₀ CFU/larva compared with 10.576 Log₁₀ CFU/larva in untreated controls, corresponding to an approximately 6.4 × 10⁵-fold reduction. Similarly, *C. auris* 1103 showed an approximately 2,800-fold reduction in CFU recovery after caspofungin treatment, decreasing from 10.627 Log₁₀ CFU/larva in untreated controls to 7.181 Log₁₀ CFU/larva in caspofungin-treated cells. This strong reduction at 24 h suggests that caspofungin pretreatment was associated with improved control of fungal burden over time. This observation is consistent with previous reports that indicated that innate immune responses to fungal pathogens intensify over time after initial recognition and immune activation (9, 14).

At 48 h post-infection, a clear strain-dependent pattern became evident. Larvae infected with caspofungin-treated *C. albicans* (*P < 0.05) and *C. auris* 1100 (****P < 0.0001) sustained significant reductions in fungal burden compared with untreated controls. In *C. albicans*, caspofungin-treated cells showed 6.058 Log₁₀ CFU/larva compared with 6.678 Log₁₀ CFU/larva in untreated controls, with an approximately fourfold reduction. *C. auris* 1100 caspofungin-treated cells showed 7.135 Log₁₀ CFU/larva compared with 9.414 Log₁₀ CFU/larva in untreated controls, corresponding to an approximately 190-fold reduction. These findings suggest that caspofungin pretreatment was associated with sustained fungal burden over time. This agrees with studies that suggest that echinocandins can enhance host-mediated fungal clearance beyond early time points in susceptible strains (8, 44). In contrast, *C. auris* 1103 showed no significant difference (ns) between caspofungin-treated cells (10.579 Log₁₀ CFU/larva) and untreated controls (10.420 Log₁₀ CFU/larva) at 48 h, despite a significant reduction at earlier time points. This pattern suggests that *C. auris* 1103 may have a strain-specific ability to persist under prolonged host pressure. This is consistent with studies reporting the viability of immune-mediated killing in *C. auris* strains (45). The difference in fungal burden between larvae infected with *C. auris* 1100 and *C. auris* 1103 at 48 h further supports the reported variability and heterogeneity of *C. auris* strains in interacting with the host immune system (17, 36, 45).

Overall, these findings show that infections with caspofungin-treated *Candida* cells were associated with reduced fungal burden *in vivo*. This reduction is consistent with increased β-(1,3)-glucan exposure and improved host-mediated clearance. However, additional mechanistic studies are needed to determine whether β-(1,3)-glucan exposure directly drives this effect in the *Galleria mellonella* model.

### Conclusion

This study demonstrates that sublethal exposure to caspofungin is associated with increased β-(1,3)-glucan exposure in multidrug-resistant *C. auris* and correlates with improved innate immune interactions. Although this phenomenon has been observed in *C. albicans* by Hasim et al. (29), our data show that caspofungin similarly promotes cell wall remodeling in *C. auris*, increasing surface-exposed β-(1,3)-glucan without substantially inhibiting fungal growth under the selected sublethal conditions. The SEM revealed changes in cell surface morphology following caspofungin treatment, consistent with disruption of the outer cell wall under sublethal conditions, although SEM does not directly identify specific cell wall components. Differences in the extent of cell wall remodeling and β-(1,3)-glucan exposure were observed between species and among *C. auris* strains, with *C. auris* exhibiting greater tolerance to caspofungin than *C. albicans*. This increased β-(1,3)-glucan exposure was associated with immune outcomes. Caspofungin-treated *C. auris* strains 1100 and 1103 exhibited greater engulfment by macrophages at 90 min, which correlated with increased surface exposure of β-glucan. This was supported by functional killing assays, which showed reduced recovery of caspofungin-treated cells at 2 and 6 h across all strains. Although increased β-(1,3)-glucan exposure correlated with increased macrophage uptake and macrophage-mediated clearance in our study, the present work does not directly establish a mechanistic relationship between glucan exposure and macrophage recognition. Additional mechanistic studies will be needed to determine the extent to which β-(1,3)-glucan exposure specifically contributes to the observed immune response, as other caspofungin-induced cell wall changes may also play a role. In *C. auris*, this increased macrophage-mediated clearance persisted through 24 h, whereas the effect diminished in *C. albicans*, consistent with known differences in fungal survival strategies.

**Fig 10:**
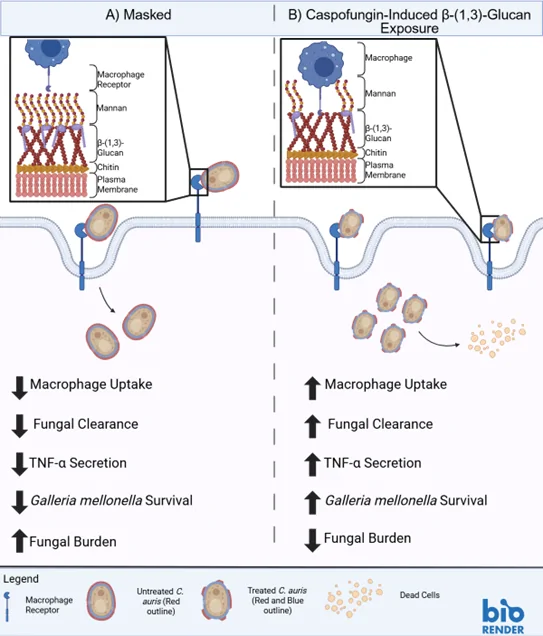
Conceptual model of caspofungin-induced cell wall remodeling and immune unmasking in *Candida auris*. Sublethal caspofungin exposure remodels the fungal cell wall, shifting cells from a masked state, with limited surface accessibility of β-(1,3)-glucan (**A**) to a state with increased β-glucan exposure (**B**). Increased β-glucan accessibility is associated with enhanced macrophage uptake, increased macrophage-mediated clearance, increased TNF-α secretion, improved *Galleria mellonella* survival, and reduced fungal burden. This figure summarizes the conceptual framework supported by the immunofluorescence, scanning electron microscopy, macrophage interaction, and *Galleria mellonella* infection assays. Created with BioRender.com.

Our findings align with the conceptual model illustrated in Fig. 10, in which caspofungin-driven remodeling transitions *Candida* cells from a state of lower β-(1,3)-glucan surface exposure (left panel) to one with increased β-(1,3)-glucan accessibility (right panel). In the untreated state, the mannan-rich outer surface restricts access to underlying β-(1,3)-glucan, limiting macrophage recognition. In contrast, caspofungin-induced remodeling increases β-glucan surface accessibility, which is associated with enhanced immune recognition and macrophage-mediated clearance.

We also observed increased TNF-α secretion in macrophages exposed to caspofungin-treated *C. albicans* and *C. auris* strain 1100, but not strain 1103, indicating strain-specific differences in macrophage cytokine responses. In the *Galleria mellonella* model, infection with caspofungin-treated *Candida* cells was associated with improved host survival and reduced fungal burden compared with untreated controls.

Together, these findings support that caspofungin-induced cell wall remodeling is associated with altered host–pathogen interactions and improved host-mediated fungal clearance. This work highlights β-(1,3)-glucan exposure as an immunologically relevant consequence of echinocandin exposure and underscores its potential importance in shaping host responses to *C. auris* infection.

### Future perspectives

While this study establishes a strong association between caspofungin-induced cell wall remodeling and enhanced immune recognition, several important questions remain. Because SEM provides surface detail without biochemical specificity, future work should combine ultrastructural imaging with biochemical assays to directly quantify changes in mannan and β-glucan architecture. Expanding the strain panel for both *C. albicans* and *C. auris* will be critical to determine how genetic and phenotypic diversity influences β-(1,3)-glucan exposure responses following echinocandin treatment.

Further investigation using receptor-blocking strategies or immune-deficient models will be necessary to define the host pathways responsible for enhanced macrophage uptake and killing. In addition, validation of these findings in vertebrate infection models will be essential to assess the relevance of drug-induced cell wall remodeling and β-(1,3)-glucan exposure under physiologically relevant conditions. Together, these future directions will help clarify how fungal cell wall remodeling shapes host–pathogen interactions and will inform the potential of leveraging cell wall remodeling pathways to improve antifungal immune responses.

## Data Availability

The data presented in this study are available on request from the corresponding author.

## SUPPLEMENTAL MATERIAL

Fig. S1 spectrum.00184-26-s0001.pdf Growth kinetics of *Candida* strains exposed to caspofungin in RPMI medium.

Fig. S2 spectrum.00184-26-s0002.pdf Quantitative ImageJ analysis of caspofungin-induced β-(1,3)-glucan-positive surface area in *Candida* species.

Table S1 spectrum.00184-26-s0003.pdf Doubling times of *Candida* strains exposed to caspofungin in RPMI medium.

ASM does not own the copyrights to Supplemental Material that may be linked to, or accessed through, an article. The authors have granted ASM a non-exclusive, world-wide license to publish the Supplemental Material files. Please contact the corresponding author directly for reuse.
