# Supplementary material for: Caspofungin-induced β-(1,3)-glucan unmasking is associated with enhanced innate immune clearance of Candida (Candidozyma) auris
Source: Microbiol Spectr. 2026 Sep 30;14(11):e00184-26. doi: 10.1128/spectrum.00184-26 (PMC13729172; doi:10.1128/spectrum.00184-26)
Supplement: Supplementary file 1 [file spectrum.00184-26-s0001.pdf]

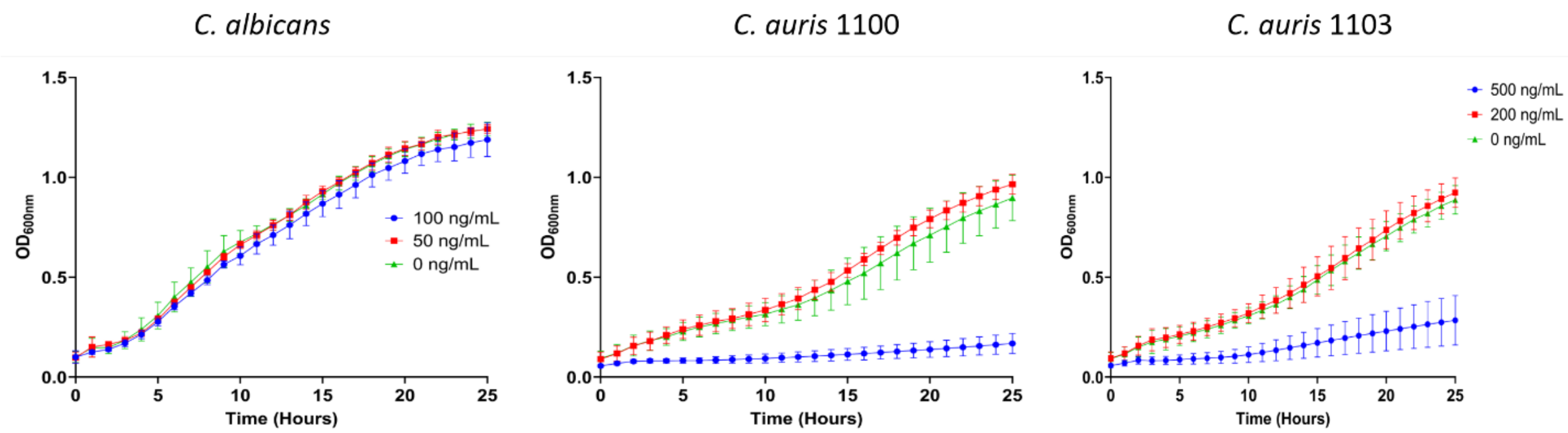

Supplementary Figure 1: Growth kinetics of *Candida* strains exposed to caspofungin in RPMI medium. *C. albicans* SC5314, *C. auris* 1100, and *C. auris* 1103 were cultured overnight in YPD, washed with PBS, and adjusted to an OD_{600} of 0.1 in RPMI medium before seeding into 96-well plates containing the indicated final caspofungin concentrations (100, 50 ng/mL for *C. albicans* and 500 and 200ng/mL for the *C. auris* strains). Each strain has a parallel untreated control. The plates were incubated at 30 °C, and the OD_{600} was measured every hour for 25 hours. At 100 ng/mL concentration, *C. albicans* growth was modestly reduced compared with the untreated control (0 ng/mL). Growth at 50 ng/mL caspofungin concentration was comparable to that of the untreated control. Both *C. auris* strains exhibited pronounced growth inhibition at 500 ng/mL, whereas growth at 200 ng/mL remained similar to the untreated control. Data are presented as mean ± SD from three independent biological replicates, each performed with three technical replicates. Doubling times were calculated from the exponential growth phase and analyzed separately (Supplementary Table 1).
