# Supplementary material for: Caspofungin-induced β-(1,3)-glucan unmasking is associated with enhanced innate immune clearance of Candida (Candidozyma) auris
Source: Microbiol Spectr. 2026 Sep 30;14(11):e00184-26. doi: 10.1128/spectrum.00184-26 (PMC13729172; doi:10.1128/spectrum.00184-26)
Supplement: Supplementary file 2 [file spectrum.00184-26-s0002.pdf]

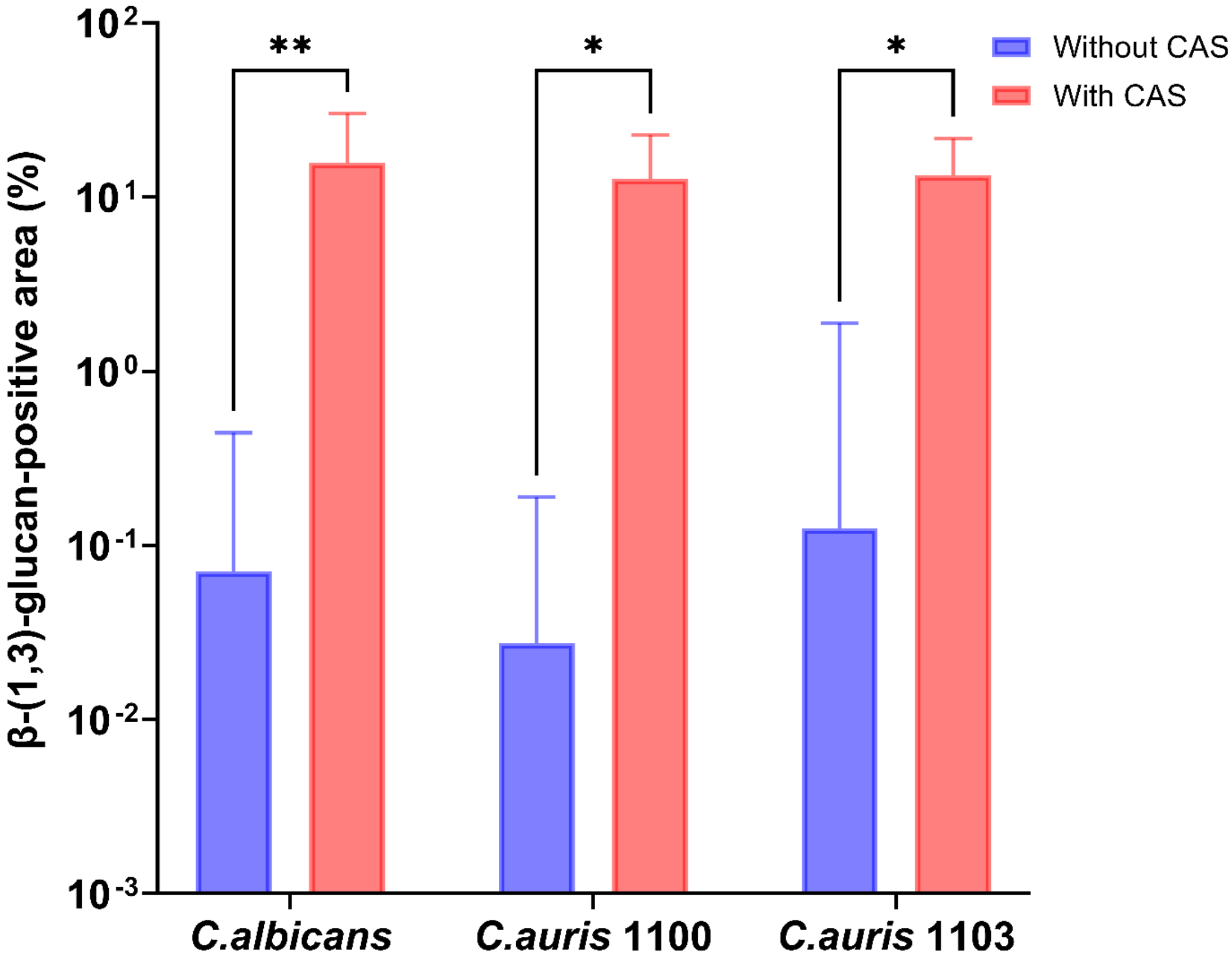

Supplementary Figure 2. Caspofungin treatment increases β-(1,3)-glucan-positive surface area in *Candida* species. β-(1,3)-glucan exposure was quantified by Cy3 staining in *C. albicans* SC5314 and *C. auris* strains 1100 and 1103 under untreated conditions (without CAS, blue) or following sublethal caspofungin treatment (with CAS, red). β-(1,3)-glucan staining was performed using a primary mouse anti-β-(1,3)-glucan antibody diluted 1:600 in PBS containing 3% BSA and 1 mM $MgCl_2$ for 90 minutes, followed by incubation with Cy3-conjugated secondary antibody diluted 1:300 in PBS containing 5% BSA and 1 mM $MgCl_2$ for 45 minutes. Quantitative fluorescence image analysis was performed in Fiji/ImageJ to determine the percentage of β-(1,3)-glucan-positive stained area from representative fluorescence images obtained from three independent biological replicates (n = 3). Bars represent mean ± SD. Caspofungin-treated cells exhibited significantly greater β-(1,3)-glucan-positive surface area compared to untreated controls within each strain, consistent with enhanced β-glucan exposure. Statistical analysis was performed using two-way ANOVA followed by Šídák's multiple comparisons test (*$p < 0.05$, **$p < 0.01$). The y-axis is displayed on a log10 scale.
