# Supplementary material for: Caspofungin-induced β-(1,3)-glucan unmasking is associated with enhanced innate immune clearance of Candida (Candidozyma) auris
Source: Microbiol Spectr. 2026 Sep 30;14(11):e00184-26. doi: 10.1128/spectrum.00184-26 (PMC13729172; doi:10.1128/spectrum.00184-26)
Supplement: Supplementary file 3 [file spectrum.00184-26-s0003.pdf]

Supplementary Table 1: Doubling time of *Candida* strains exposed to caspofungin in RPMI medium.

***Candida* Strains**	**Caspofungin Concentration (ng/mL)**	**Mean Doubling Time (h ± SE)**	**Adjusted p-value vs Control**
	0	3.408 ± 0.103	-
C. albicans	50	3.276 ± 0.022	0.2626
	100	3.668 ± 0.136	0.4034
	0	4.473 ± 0.310	-
C. auris 1100	200	4.438 ± 0.303	0.9961
	500	10.616 ± 1.206	0.0007
	0	4.828 ± 0.071	-
C. auris 1103	200	4.771 ± 0.229	0.9641
	500	8.315 ± 0.760	0.0016

Values are presented as mean doubling time in hours ± SE from three independent biological replicates. Adjusted p-values represent comparisons with untreated controls within each strain.
